# Relationship Between Vaccination and Immune Thrombotic Thrombocytopenia: Coincidental or Causal?

**DOI:** 10.7759/cureus.28560

**Published:** 2022-08-29

**Authors:** Pallavi Yelne, Ruchita Kabra, Swapneel Mathurkar, Shilpa A Gaidhane, Sourya Acharya, Sunil Kumar

**Affiliations:** 1 Medicine, Jawaharlal Nehru Medical College, Datta Meghe Institute of Medical Sciences, Wardha, IND; 2 Internal Medicine, Jawaharlal Nehru Medical College, Datta Meghe Institute of Medical Sciences, Wardha, IND; 3 Ophthalmology, Jawaharlal Nehru Medical College, Datta Meghe Institute of Medical Sciences, Wardha, IND; 4 Epidemiology and Public Health, Jawaharlal Nehru Medical College, Datta Meghe Institute of Medical Sciences, Wardha, IND

**Keywords:** covid-19 vaccination, venous sinus thrombosis, venous thrombosis, prothrombotic, vaccine

## Abstract

Vaccine-induced immune thrombotic thrombocytopenia (VITT) is a complication developed in patients due to vaccination. High-risk factors like a prothrombotic state predispose such a condition. Due to the increase in vaccinations after the coronavirus 2019 (COVID-19) pandemic, the predisposition to risk factors has increased. Hence, complications occur at a very young age. This case report is of a young male who developed venous sinus thrombosis post the COVID-19 vaccination and was diagnosed and treated promptly.

## Introduction

Vaccine-induced immune thrombotic thrombocytopenia (VITT) is a syndrome that occurs in patients who develop prothrombotic conditions due to vaccinations. VITT is similar to the phenomenon of heparin-induced thrombocytopenia (HIT) Type II [1]. The AstraZeneca (ChAdOx1 nCov-19) vaccine has been widely used to vaccinate the Indian population and has shown a good immune response as shown by different studies [2]. However, there are a few cases of VITT reported in the literature secondary to AstraZeneca vaccine injection. Few of them have developed it in the form of venous sinus thrombosis [3]. This case report presents a 25-year-old male patient who received the coronavirus 2019 (COVID-19) vaccine and, two weeks later, developed sagittal venous sinus thrombosis. He was treated with anticoagulants and not with steroids.

## Case presentation

A 25-year-old male with no medical history came to the outpatient clinic with complaints of headache and vomiting for two days. He had four to five episodes of non-bilious, non-projectile vomiting on the day of admission, which was associated with nausea and contained food particles. The patient received the first dose of ChAdOx1 nCov-19 vaccine (AstraZeneca) 17 days back. His vitals were stable on admission. On central nervous examination, there was bilateral lower limb weakness, and power in both lower limbs was 3/5. The remaining neurological and cranial nerve examination was normal. The rest of the physical examination was unremarkable. He was admitted and all routine investigations were done as shown in Table 1. His headache was severe and was not responding to non-steroidal anti-inflammatory drugs (NSAIDs).

**Table 1:** Patient’s blood investigations at the time of admission SGOT: serum glutamic-oxaloacetic transaminase; SGPT: serum glutamic pyruvic transaminase; PT-INR: prothrombin time-international normalized ratio; ANA: antinuclear antibody; RBS: random blood sugar; TSH: thyroid stimulating hormone

Investigation	Patient’s value	Normal Reference
Hemoglobin	13.7gm/dl	12-14gm/dl
WBC Count	11,200/cu mm	6000-11,000/cu mm
Platelet Count	85,000/cu mm	1,50,000-4,50,000/cu mm
SGOT	34U/L	<50U/L
SGPT	25U/L	17-50UL
Serum Urea	32 mg/dl	9-20mg/dl
Serum Creatinine	1.1mg/dl	0.6-1.2mg/dl
PT-INR	1.18	<1.2
ANA	NEGATIVE	<0.9
RBS		
Vitamin B12	432 pg/ml	240-930 pg/ml
TSH	1.2mIU/ML	0.46-4.6 mIU/ML

**Figure 1:**
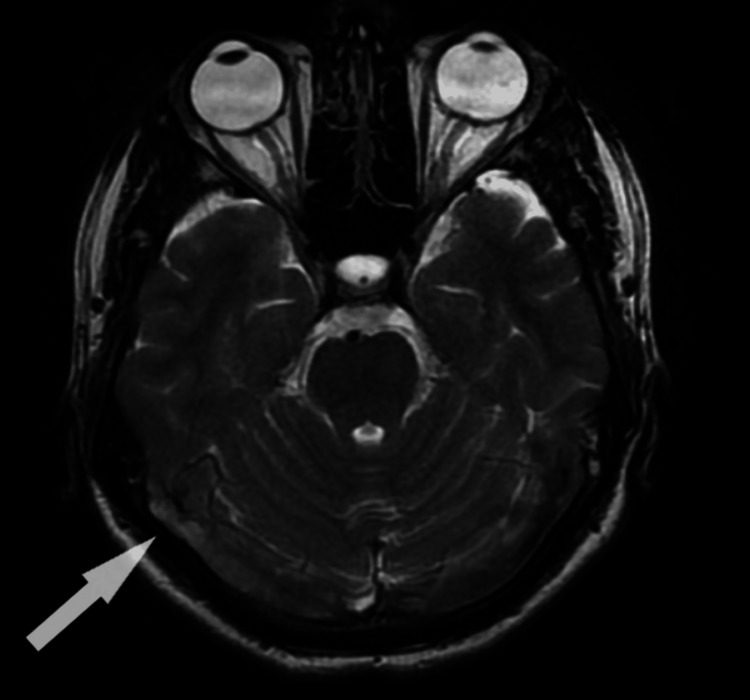
MRI Brain showing hypointense lesion in T2 weighted image (arrow)

Magnetic resonance imaging (MRI) brain with magnetic resonance venography (MRV) showed the absence of flow void on T2 weighted image along with isointense lesion on T1 weighted image and hyperintense lesion on T2 weighted image suggesting superior sagittal sinus, straight sinus, and bilateral transverse and sigmoid sinus thrombosis as shown in Figure 1. An ophthalmic examination was done in view of persistent headache suggestive of papilledema and was confirmed on optical coherence tomography (OCT) by thickening of retinal nerve fibers.

His thrombophilia profile, coagulation profile was positive for anti PF4 antibodies titre; hence, a diagnosis of VITT was made. As soon as the diagnosis was confirmed, he was started on injection mannitol 350 ml intravenous stat followed by 100 ml intravenous thrice a day. Considering cerebral sinus venous thrombosis (CSVT), a subcutaneous injection of low molecular weight heparin 6 mg twice a day was given. His lower limb weakness improved to a power of 5/5 over the duration of seven days. He was also started on injectable anti-convulsant levetiracetam 1 gram IV stat followed by 500 mg twice a day so as to prevent seizures. Other supportive management included tablet naproxen + domperidone for headache, injection ondansetron 8 mg three times a day, and injection pantoprazole 40 mg once a day. He was shifted to oral anticoagulants after three days of injectable anticoagulants, i.e., tablet rivaroxaban 15 mg twice a day. The patient became asymptomatic after treatment and was later discharged with no focal neurological deficit. On follow-up, the patient was doing well, repeat MRI Brain with MRV was done and was suggestive of resolution of venous thrombosis. A repeat ophthalmic examination was done along with OCT, which showed resolution of papilledema and the patient is doing well.

## Discussion

The ChAdOx1 nCov-19 vaccination produced a positive immunogenic response in all age groups, with no major side effects linked to the immunization [4]. Since then, other international RCTs have all shown that the ChAdOx1 nCov19 vaccine has an acceptable safety profile and it has been given to the majority of the Indian population as per the vaccination program [5]. Some clinicians believe that patients who develop venous sinus thrombosis after receiving the ChAdOx1 nCov19 vaccination have the same risk factors as the general population [6]. However, given the relatively young population groups affected by thrombotic events, a pure coincidental explanation is difficult to accept [6].

This is especially true when the patients' serological profiles are consistently aberrant. Low platelet count was a consistent finding in patients who have had thrombotic problems as a result of the ChAdOx1 nCov-19 vaccination [7]. Also, some patients' blood profiles showed high d dimer levels, which was normal in our patient. Heparin-induced thrombocytopenia is caused by platelet-activating antibodies mostly PF4, which appear in the blood after the administration of heparin to patients. These patients are usually seen in a prothrombotic state and develop complications [8,9].

It's also noticed that the same HIT phenomena were observed in patients even when heparin hasn't been given. This has been discovered in cases of bacterial and viral infections as well as in postoperative surgical patients [8]. The term "autoimmune HIT" has been coined to describe such spontaneous occurrences of HIT [10]. VITT is an example of autoimmune HIT that is becoming more common among individuals that have taken the ChAdOx1 nCov-19 vaccine. AntiPF4 antibodies are linked to higher levels of platelet activation in these patients' serum, which shows an exaggerated reaction on the PF4-heparin enzyme-linked immunosorbent assay (ELISA) [7].

One possible reason for platelet activation, according to Greinacher et al., is free DNA within the ChAdOx1 nCov-19 vaccine. Other vaccines have not been examined for free DNA, but it has been observed that only patients who have received the ChAdOx1 nCov-19 vaccine have shown VITT [7].

Management

Immunomodulation with high-potency glucocorticoids and intravenous immune globulin (IVIG) is an important part of the acute therapy of VITT patients [9]. Prompt treatment with such approaches has been found to efficiently improve platelet counts, lowering the risk of hemorrhagic consequences. Blocking FcRIIA receptors on platelets could be one of the ways IVIG works. This, like traditional HIT, can directly down-regulate platelet activation. 

Plasma exchange may be done in a critical care situation to temporarily reduce the titer of anti-PF4 antibodies in treatment-resistant instances [6]. Our patient showed great recovery after steroid treatment; hence, plasma exchange was not needed. When it comes to preventing venous thromboembolic events, most clinicians agree that non-heparin anticoagulants should be used instead of heparin. Platelet transfusions should also be avoided in individuals with VITT since they raise the risk of thrombotic events considerably.

## Conclusions

Post-vaccination thrombotic thrombocytopenia is a life-threatening complication if not identified early. Post vaccination, one should look for red flag symptoms. Signs like recent-onset headache, blurring of vision, blackouts, giddiness, etc. should be evaluated thoroughly to identify CVST. Early diagnosis of such a serious condition can be life-saving.
